# 3D-CFD Modeling of Hollow-Fiber Membrane Contactor for CO_2_ Absorption Using MEA Solution

**DOI:** 10.3390/membranes14040086

**Published:** 2024-04-09

**Authors:** Alexandru-Constantin Bozonc, Vlad-Cristian Sandu, Calin-Cristian Cormos, Ana-Maria Cormos

**Affiliations:** Faculty of Chemistry and Chemical Engineering, Babeș-Bolyai University, Arany Janos 11, 400028 Cluj-Napoca, Romania; alexandru.bozonc@stud.ubbcluj.ro (A.-C.B.); vlad.sandu@ubbcluj.ro (V.-C.S.); calin.cormos@ubbcluj.ro (C.-C.C.)

**Keywords:** CO_2_ capture process, HFMC, absorption, 3D-CFD modeling

## Abstract

Membrane technology is considered an innovative and promising approach due to its flexibility and low energy consumption. In this work, a comprehensive 3D-CFD model of the Hollow-Fiber Membrane Contactor (HFMC) system for CO_2_ capture into aqueous MEA solution, considering a counter-current fluid flow, was developed and validated with experimental data. Two different flow arrangements were considered for the gas mixture and liquid solution inside the HFMC module. The simulation results showed that the CO_2_ absorption efficiency was considerably higher when the gas mixture was channeled through the membranes and the liquid phase flowed externally between the membranes, across a wide range of gas and liquid flow rates. Sensitivity studies were performed in order to determine the optimal CO_2_ capture process parameters under different operating conditions (flow rates/flow velocities and concentrations) and HFMC geometrical characteristics (e.g., porosity, diameter, and thickness of membranes). It was found that increasing the membrane radius, while maintaining a constant thickness, positively influenced the efficiency of CO_2_ absorption due to the higher mass transfer area and residence time. Conversely, higher membrane thickness resulted in higher mass transfer resistance. The optimal membrane thickness was also investigated for various inner fiber diameters, resulting in a thickness of 0.2 mm as optimal for a fiber inner radius of 0.225 mm. Additionally, a significant improvement in CO_2_ capture efficiency was observed when increasing membrane porosity to values below 0.2, at which point the increase dampened considerably. The best HFMC configuration involved a combination of low porosity, moderate thickness, and large fiber inner diameter, with gas flow occurring within the fiber membranes.

## 1. Introduction

Global warming has become a major issue in recent years due to the steady rise in greenhouse gas emissions. Carbon dioxide (CO_2_) accounts for about 76% of total greenhouse gas emissions [1]. The rapid rate of growth of atmospheric CO_2_ concentrations, from 280 ppm in 1850 to 418 ppm in 2022 [2], has driven a substantial increase in research concerning mitigation of CO_2_ emissions. Carbon capture, utilization, and storage (CCUS) is a technology used to reduce CO_2_ emissions and diminish the global warming phenomenon [3,4]. Among CCUS technologies, post-combustion technologies are the most convenient to implement on an industrial scale. The CO_2_ separation process takes place after the combustion of fossil fuels in power plants or after the generation of products for other industrial processes (i.e., steel and iron production). As such, a complete overhaul of the existing industrial plant is not necessary and only the installation of an additional unit for CO_2_ capture is required. There are various techniques applied in CO_2_ capture, such as absorption, adsorption, and membrane-based and cryogenic separation [5,6,7]. Considering the advantages and disadvantages of these CO_2_ capture methods, the post-combustion gas–liquid absorption process demonstrates enhanced viability due to its high absorption capacity and low energy consumption [8,9], as well its ability to be integrated into existing power plants by introducing the CO_2_ capture stage.

In the gas–liquid absorption process, the absorption solution represents a crucial component as the nature of the absorbent dictates the speed of absorption and also plays an important role in the dimensions of the absorber, with a faster reaction leading to a reduction in unit size. The most commonly used chemical solvents in the absorption of CO_2_ are monoethanolamine (MEA), diethanolamine (DEA), methyldiethanolamine (MDEA), ammonia, and sodium hydroxide [2]. Among all alkanolamines, MEA is the most widely used in industrial processes, primarily due to its very fast reaction with CO_2_ and high CO_2_ absorption capacity. These properties enable the minimization of the absorption column dimensions and reduction of the liquid flow rate, inherently lowering the energy consumption associated with transportation, heating, and cooling. In addition, MEA has a low price and high water solubility. The disadvantages of MEA include high viscosity at high concentrations, high corrosion, and high regeneration energy [2,10].

Compared to conventional packed-bed columns, the use of hollow-fiber membrane contactors (HFMC) for CO_2_ capture provides a considerable number of advantages, such as: (i) a significantly larger mass transfer area per unit volume, resulting in a smaller absorber; (ii) membrane systems are highly modular, so scale-up is simpler; (iii) the mass transfer area is constant and independent of hydrodynamic conditions; (iv) membrane systems are easier to replace or repair; (v) more environmental friendly; (vi) due to the physical separation between the two phases, the operating problems related to foaming, flooding, channeling, entrainment, and formation of emulsions are avoided; (vii) reduced loss of solvent; (viii) lower operating and investment costs; (ix) fluid flow rates are not limited to each other [10,11,12,13,14]. Given these advantages, the use of HFMC shows great potential for intensification of the gas–liquid absorption CO_2_ capture process. However, there are also drawbacks to using HFMC for carbon capture, such as: (i) membranes introduce an additional mass transfer resistance for CO_2_; (ii) transfer resistance increases significantly over time due to membrane watering; (iii) due to the short lifetime, the membranes need to be replaced periodically [2,10,11,12].

**Figure 1:**
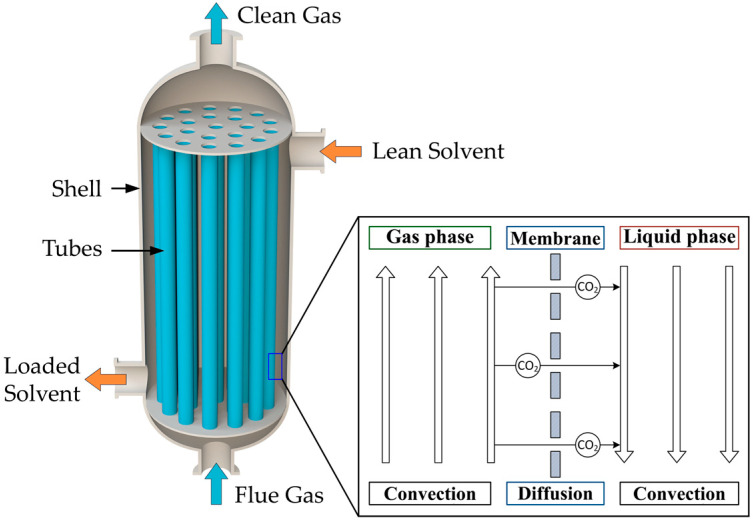
Schematic representation of the HFMC for CO_2_ absorption.

A schematic representation of the HFMC is presented in Figure 1, along with a representation of the CO_2_ transfer from the gas phase into the liquid phase through the membrane pores.

The HFMC module consists of a large number of hollow cylindrical membranes inside the shell compartment. There are three distinct regions in the HFMC: the space inside the membranes—the tube side, the hollow-fiber porous membranes, and the space between the membranes—the shell compartment. As the gas mixture flows inside the membrane tubes and the liquid absorbent solution flows between them in a counter-current arrangement, the membranes physically separate the two phases. The CO_2_ diffuses from the gas phase through the membrane pores and is absorbed into the liquid solution, where it reacts with the MEA to form a stable compound. The resulting product will then be transported to the desorption column in order to obtain a high purity stream of CO_2_ and regenerate the MEA solution (which is subsequently recycled to the absorption stage).

Several models for the capture of CO_2_ using HFMC are presented in the literature, divided into one-dimensional (1D), two-dimensional (2D), and three-dimensional (3D) spatial models, based on the process-simplifying assumptions that have been made [15]. For the 1D models, the angular and axial variations of the process parameters are not accounted for, and only the variation over one dimension is considered. For these models, the radial direction is the one of interest, considering a series resistance for CO_2_ mass transfer (i.e., resistance of gas, porous membrane, and liquid solution). Based on the two-film theory, the CO_2_ will diffuse through the gas film, through the membrane pores and into the liquid solution. The mass transfer for each diffusion step can be represented with partial mass transfer coefficients, which can then be used to determine a global mass transfer coefficient [16]. In addition, the chemical reaction can also be integrated by using the enhancement factor brought by the reaction [17]. The 1D models available in the literature also consider different solvents for the absorption process and whether the membranes are wetted or not [18,19,20,21,22,23]. The 2D models suppose a single fiber inside the HFMC, considering the variation of parameters over the axial and radial directions occurring within the diffusive and convective transport mechanisms. The CFD modeling consists of a 2D axisymmetric spatial component, thus allowing the representation of a 3D geometry for one hollow-fiber membrane by revolving the 2D results around the axis of symmetry [24,25,26,27,28,29,30,31,32]. The 2D models provide a better understanding of the CO_2_ absorption process using HFMC compared to the 1D models. However, the 2D models are limited to obtaining results for only a single fiber. The development of a 3D model is necessary to achieve higher accuracy and consider phenomena that are not captured in the 1D and 2D models, such as: non-uniform distribution of the fluids, non-uniform properties of the fluids and membranes, interaction between the fibers, etc. The main limitation of 2D models, compared to the 3D model developed in this work, is that they are axisymmetric and use the Happel’s approximation model to determine the effective radius of the shell around one membrane. Additionally, the 3D model also considers the fluid distribution around the shell wall, an effect that is also overlooked in the available 2D models. A drawback to the increased complexity enabled by the 3D models is the requirement to use more computational resources in order to solve the governing equations. There is a limited number of 3D models currently available in the literature [33,34,35], indicating substantial potential for the development of 3D models for membrane-based CO_2_ capture [15].

In this work, a comprehensive computational fluid dynamics (CFD) 3D model of the HFMC system for CO_2_ capture considering an aqueous MEA solution and counter-current fluid flow is developed and validated with experimental data. The novelty of this work lies in the modeling and simulation of the full 3D HFMC geometry for CO_2_ capture. The effect of different operating conditions (flow rates/flow velocities and concentrations) and HFMC geometrical characteristics (porosity, number, inner diameter, thickness, and length of fiber membranes) on CO_2_ capture process parameters is investigated. In addition, the impact of how the fluids flow inside the fiber membrane module is evaluated.

## 2. Mathematical Model Approach

**Figure 2:**
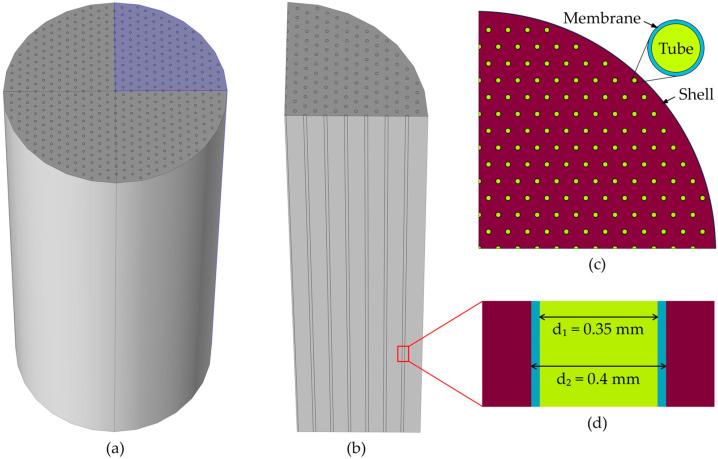
(**a**) 3D HFMC geometry representation, (**b**) quarter of the HFMC 3D representation, (**c**) 2D horizontal section representation of the distribution of membranes in the shell of the HFMC, (**d**) 2D vertical section representation of a membrane.

The CFD model for CO_2_ absorption was developed using COMSOL Multiphysics, implementing the HFMC geometry represented in Figure 2. In Figure 2a, the entire HFMC geometry is represented, containing 510 membranes arranged symmetrically under a hexagonal distribution inside the shell. In order to reduce the simulation time and the computational resources needed, the complete HFMC module is sliced into four symmetrical parts (Figure 2b), resulting in only a quarter of the HFMC module being simulated. A 2D horizontal section of the simulated geometry is represented in Figure 2c, where the hexagonal symmetrical distribution can be better observed, as well as the distinct sections of the geometry (i.e., tubes—inside the membranes, membranes, and the shell compartment). A section through one of the membranes is represented in Figure 2d. The gas mixture flows inside the membranes (i.e., tubes) and the liquid MEA solution flows between the membranes (i.e., shell) in a counter-current arrangement.

**Figure 3:**
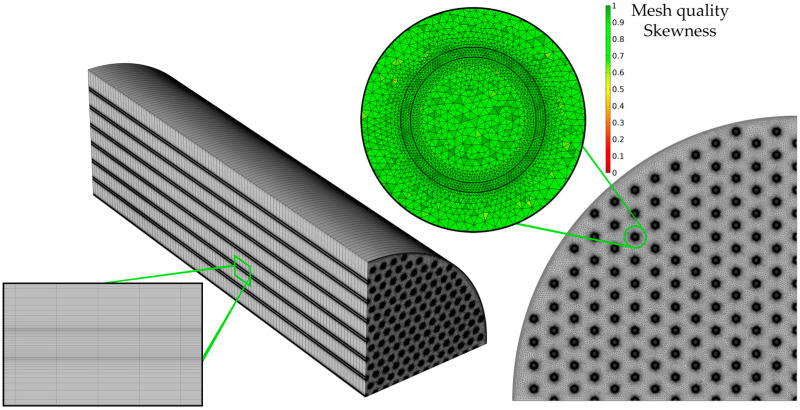
Mesh of the HFMC and quality of the mesh.

The mesh used to spatially discretize the HFMC in the CFD model is represented in Figure 3.

The geometry was sliced in 100 equal pieces. The mesh for each slice (presented in the right corner of Figure 3) consists of a triangular mesh, with an increase in element density in the regions of interest: inside and around the membranes, as this is where CO_2_ from the gas phase diffuses through the membrane, is absorbed in the liquid, and reacts with the MEA. In addition, the mesh quality is presented in terms of element skewness, with green representing the highest quality. The mesh was sufficiently refined until mesh convergence was reached and the model predicted mesh-independent results. The number of mesh elements for a single fiber membrane was 130,800 (the elements for all membranes in the implemented geometry was 16,731,100), 166,000 for discretization of a tube (all tubes required 20,979,300 elements), and 34,107,600 for the shell, with a total average quality of about 0.85.

The HFMC characteristics are presented in Table 1, along with the process operating conditions, which are represented in Table 2.

In order to develop the CFD model for CO_2_ absorption using HFMC, the following assumptions have been made:

- Isothermal conditions for the fluids;
- The membranes are operating in non-wet condition;
- The CO_2_ solubility in aqueous MEA solution is calculated with Henry’s law;
- Fully developed laminar flow for both fluid phases (i.e., gas and liquid).

The 3D CFD model of the HFMC was implemented in COMSOL Multiphysics 6.1, using a 64-bit operating system, with two Intel(R) Xeon(R) Platinum 8168 CPU @ 2.70 GHz and 512 GB of RAM.

### 2.1. Continuity Equation

The equations are solved in dynamic conditions, therefore the continuity equations (i.e., conservation of mass) for both phases are:

(1) $$\frac{\partial }{\partial t}\left(\rho_{i}\right)+\nabla \cdot \left(\rho_{i}u_{i}\right)=0$$

### 2.2. Navier–Stokes Equations—Tubes and Shell

The flow regimes for the gas mixture inside the tubes and the liquid solution inside the shell are determined by calculating the Reynolds numbers with values of 18.4 and 44, respectively, indicating laminar flow types for both phases. In order to solve the laminar flows inside the membranes and surrounding them, the model used the continuity equations (Equation (1)) and the Navier–Stokes equations (i.e., conservation of momentum) (Equation (2)):

(2) $$\rho_{i}\frac{\partial u_{i}}{\partial t}+\rho_{i}\left(u_{i}\cdot \nabla \right)u_{i}=\nabla \cdot \left(-p_{i}I+\tau_{i}\right)$$

where $\tau_{i}$ is the viscous stress tensor, calculated using (Equation (3)):

(3) $$\tau_{i}=\mu_{i}\left(\nabla u_{i}+{\left(\nabla u_{i}\right)}^{T}\right)-\frac{2}{3}\mu_{i}\left(\nabla \cdot u_{i}\right)I$$

### 2.3. Species Transport—Tubes

In the gas phase, there is no chemical reaction, therefore the mass transfer of species inside the membranes is described only by convection and diffusion, considering an isotropic diffusion (Equation (4)):

(4) $$\frac{\partial c_{j}}{\partial t}+\nabla \cdot \left(-D_{j,G}\nabla c_{j}\right)+u\cdot \nabla c_{j}=0$$

Equation (5), known as the Graetz–Lévêque solution [10], is used to predict the partial mass transfer coefficient of CO_2_ inside the membranes (i.e., tube side):

(5) $$Sh_{tube}=\frac{k_{{\mathrm{CO}}_{2},g}d_{1}}{D_{{\mathrm{CO}}_{2},g}}=1.62{\left(\frac{d_{1}}{L}Re\right)}^{0.33}$$

### 2.4. Species Transport—Membranes

The mass transfer in the hydrophobic microporous membranes is represented by the convection and diffusion of gas components through the membrane pores, which are considered to be filled only with gas as the membrane functions in a non-wet state (Equation (6)):

(6) $$\varepsilon_{mem}\frac{\partial c_{j}}{\partial t}+\nabla \cdot \left(-D_{j,mem}\nabla c_{j}\right)+u\cdot \nabla c_{j}=0$$

The effective diffusion coefficient of component *j* through the membrane pores $D_{j,mem}$ is calculated considering the membrane porosity $\varepsilon_{mem}$ and tortuosity $\tau_{mem}$ (Equation (7)), assuming an isotropic diffusion:

(7) $$D_{j,mem}=\frac{\varepsilon_{mem}}{\tau_{mem}}D_{j,G}$$

The partial mass transfer coefficient of CO_2_ through the membrane is determined using Equation (8) [26]:

(8) $$k_{{\mathrm{CO}}_{2},mem}=\frac{D_{{\mathrm{CO}}_{2},mem}}{\delta_{mem}}$$

### 2.5. Species Transport—Shell

Inside the shell compartment, the CO_2_ absorbed from the gas phase into the liquid solution reacts with MEA. Therefore, the equation that describes mass transfer inside the shell (Equation (9)) also contains the chemical reaction rate $R_{j}$, together with terms for diffusion and convection:

(9) $$\frac{\partial c_{j}}{\partial t}+\nabla \cdot \left(-D_{j,L}\nabla c_{j}\right)+u\cdot \nabla c_{j}=R_{j}$$

According to Yang and Cussler in their work on gas absorption and stripping, the partial mass transfer coefficient of CO_2_ inside the shell side, where fluid flow is parallel to the fibers, can be calculated with Equation (10) (0.5 < *Re* < 500) [36]:

(10) $$Sh_{shell}=\frac{k_{{\mathrm{CO}}_{2},l}d_{h}}{D_{{\mathrm{CO}}_{2},l}}=1.25{\left(Re\frac{d_{h}}{L}\right)}^{0.93}Sc^{0.33}$$

According to the double-film theory, the overall resistance of mass transfer is the sum of the resistances of all layers through which the CO_2_ diffuses. The resistance of each layer can be measured by calculating the inverse of the partial mass transfer coefficient of CO_2_, indicating the difficulty for the CO_2_ molecules to diffuse through that particular layer (fluid or material). Therefore, the overall mass transfer coefficient of CO_2_ based on the liquid phase can be calculated using the sum of the three mass transfer resistances inside of the HFMC: liquid ($1/k_{{\mathrm{CO}}_{2},l}$), membrane ($1/k_{{\mathrm{CO}}_{2},m}$), and gas ($1/k_{{\mathrm{CO}}_{2},g}$), while also accounting for the dimensions of the system and the CO_2_ solubility in the absorbent solution, as shown in Equation (11) [37]:

(11) $$\frac{1}{K_{L}d_{2}}=\frac{1}{k_{{\mathrm{CO}}_{2},l}d_{2}}+\frac{1}{k_{{\mathrm{CO}}_{2},m}H_{{\mathrm{CO}}_{2}}d_{lm}}+\frac{1}{k_{{\mathrm{CO}}_{2},g}H_{{\mathrm{CO}}_{2}}d_{1}}$$

The CO_2_ mass transfer flux from the gas phase to the liquid phase is calculated based on the overall mass transfer coefficient of CO_2_, the concentration gradient at the gas–liquid interface, and the absorption enhancement factor *E* given by the chemical reaction (Equation (12)) [23]:

(12) $$J_{{\mathrm{CO}}_{2}}=E\cdot K_{L}\cdot \left(\frac{C_{{\mathrm{CO}}_{2}}^{g}}{H_{{\mathrm{CO}}_{2}}}-C_{{\mathrm{CO}}_{2}}^{L}\right)$$

The reaction of CO_2_ and MEA, which takes place within the liquid phase located in the shell compartment of the HFMC, is described by the following reaction rate expression [26]:

(13) $$R_{{\mathrm{CO}}_{2}-\mathrm{MEA}}=\frac{10^{\left(10.99-2152/T_{L}\right)}}{1000}C_{{\mathrm{CO}}_{2}}^{L}C_{\mathrm{MEA}}^{L}$$

The efficiency of the absorption process of CO_2_ using HFMC in an MEA solution is calculated using Equation (14), considering the variation in CO_2_ concentration in the flue gas and the clean gas:

(14) $${\mathrm{CO}}_{2}\;\mathrm{capture}\;\mathrm{rate}=\left(1-\frac{C_{{\mathrm{CO}}_{2},outlet}}{C_{{\mathrm{CO}}_{2},inlet}}\right)\cdot 100$$

## 3. Results and Discussion

The CFD model for the CO_2_ absorption process in an MEA solution using HFMC was implemented with the membrane module characteristics presented in Table 1 at different operating conditions (Table 2).

### 3.1. Model Validation

**Figure 4:**
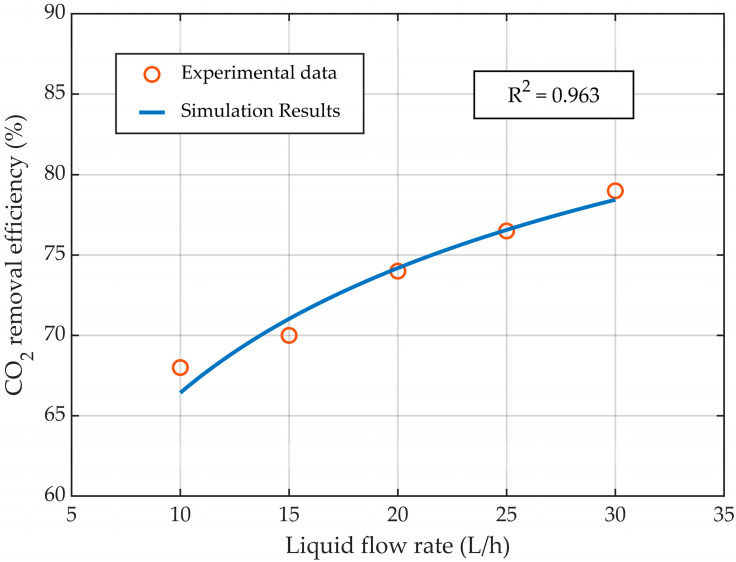
CO_2_ capture rate profile at different liquid flow rates, at a constant gas flow rate of 2 L/min.

**Figure 5:**
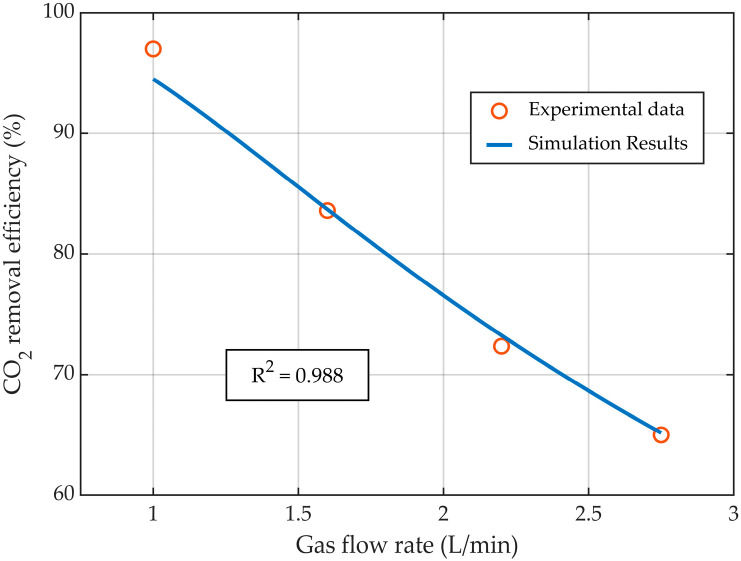
CO_2_ capture rate profile at different gas flow rates, at a constant liquid flow rate of 25 L/h.

**Figure 6:**
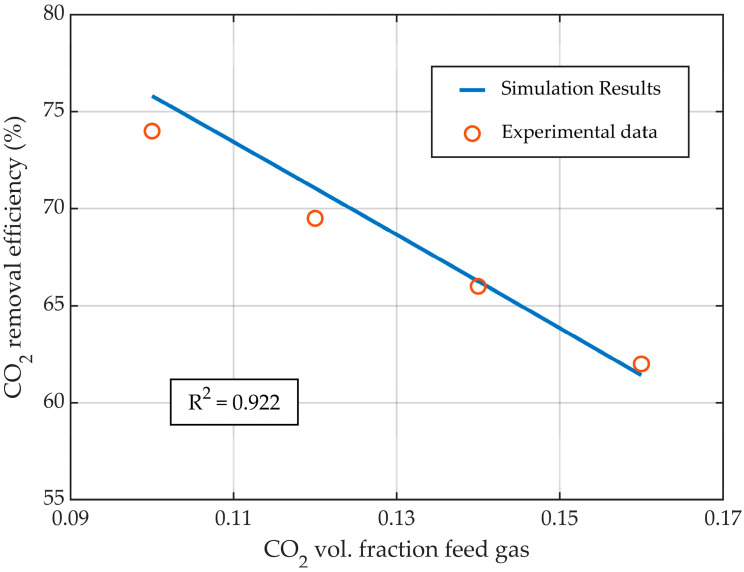
CO_2_ capture rate profile at different inflow CO_2_ concentration in gas phase, at constant gas (2 L/min) and liquid flow rates (25 L/h).

The CFD model of the HFMC for CO_2_ absorption was validated with experimental data published in the literature [31]. The validation was performed by comparing the experimental data with the simulation results in terms of CO_2_ capture rate (Equation (14)), at various liquid flow rates (Figure 4), gas flow rates (Figure 5), and CO_2_ gas inflow concentrations (Figure 6). Overall, the simulation results showed excellent agreement with the experimental data, with R^2^ > 0.922, demonstrating that the developed CFD model represented the process of CO_2_ absorption in an MEA solution using HFMC with high accuracy.

By increasing the liquid flow rate and maintaining a constant gas flow rate, the absorption efficiency of CO_2_ showed an increase (Figure 4) due to the rise in the amount of MEA that flowed inside the HFMC and reacted with the absorbed CO_2_. Comparing the experimental data with the simulation results, a very good correlation was observed, with an R^2^ = 0.963. The CO_2_ removal efficiency was increased from about 66.5% to nearly 79% for values of the liquid flow rate ranging from 10 L/h to 30 L/h, respectively.

While maintaining the liquid flow rate constant at 25 L/h, the increase in the gas flow rate determined a decrease in CO_2_ capture rate (Figure 5) due to the reduction in the CO_2_ residence time inside the HFMC. The experimental data and the simulation results exhibited a very good correlation, with an R^2^ value of nearly 0.99. By increasing the gas flow rate from 1 L/min to 2.75 L/min, the CO_2_ absorption efficiency decreased from 94.5% to nearly 65%, respectively.

The increase in the concentration of CO_2_ in the inflow gas led to a decrease in the absorption efficiency of CO_2_ (Figure 6), while maintaining constant gas and liquid flow rates, due to the increase in the amount of CO_2_ in the gas phase. The simulation results were in good agreement with the experimental data, with an R^2^ = 0.922. Changing the CO_2_ concentration in the inflow gas from 0.1 to 0.16 vol. fraction, the CO_2_ removal efficiency was decreased from nearly 76% to 61.5%, respectively.

### 3.2. Velocity Profiles

**Figure 7:**
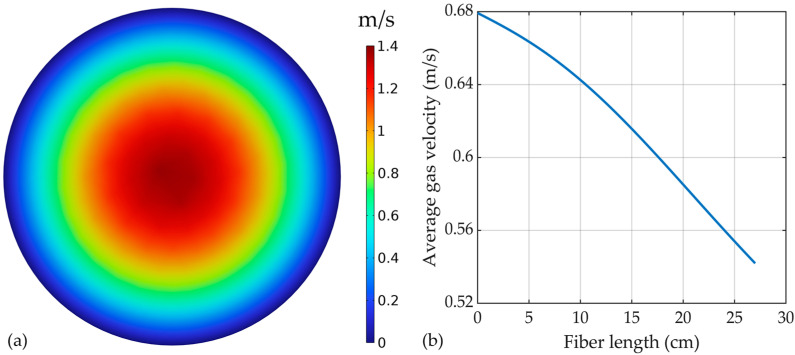
(**a**) Gas mixture velocity profile inside the tubes, (**b**) average gas velocity profile along the HFMC length. Gas flow rate of 2 L/min.

The gas velocity inside the membranes, within the tubes, is represented in Figure 7a. The velocity profile predicted by the model showed a typical laminar shape for fluid flow inside a tube. 

The maximum velocity was observed in the center of the tubes, at around 1.37 m/s (*Re* = 14), while a decreasing velocity was seen closer to the inside membrane wall, with values of zero at the contact with the membrane, due to the friction with the membrane wall and the no-slip boundary conditions assumed at the wall. The average gas velocity profile along the HFMC module is presented in Figure 7b. A reduction in the gas velocity from around 0.68 m/s at the inlet to 0.542 m/s at the outlet of the HFMC module.

**Figure 8:**
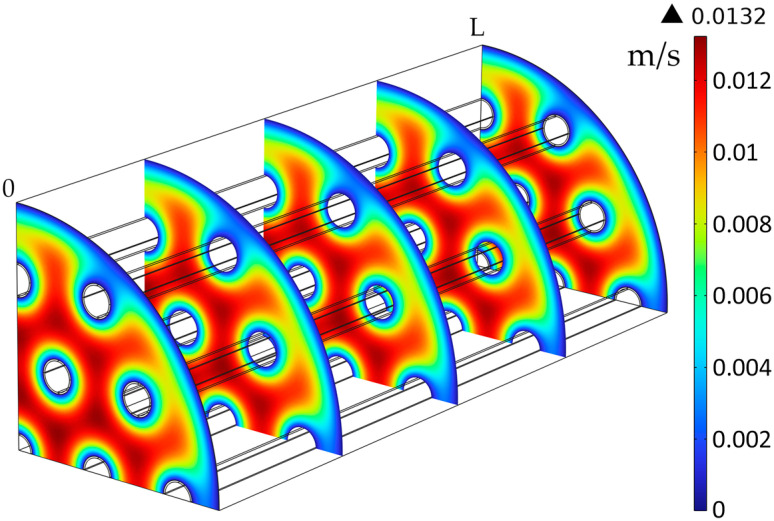
Liquid velocity profile in the shell compartment, Q_L_ = 25 L/h.

The solution for the liquid phase velocity profile surrounding the membrane tubes is represented in Figure 8, with the maximum velocity of the liquid seen between the membranes, at around 0.0132 m/s (*Re* = 37). The liquid velocity was decreasing closer to the membranes and module walls, with a velocity near zero where the friction between the fluid and the walls was highest at the edges.

### 3.3. Concentration Profiles

**Figure 9:**
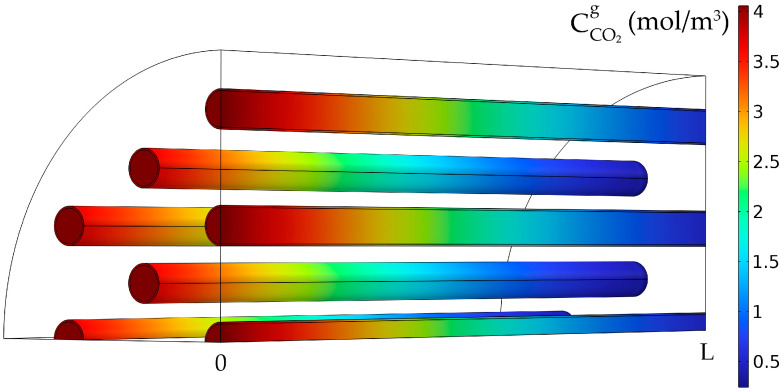
CO_2_ gas concentration in the tubes. $C_{\mathrm{MEA},0}$ = 818 mol/m^3^ (5 wt%), $C_{{\mathrm{CO}}_{2},0}$ = 4.06 mol/m^3^ (10 vol%), Q_g_ = 2 L/min, Q_L_ = 25 L/h.

The CO_2_ concentration in the gas phase inside the membranes (tubes) decreased over the length of the membranes, from about 4.06 mol/m^3^ at the tube inlet to nearly 0.62 mol/m^3^ at the outlet, resulting in a CO_2_ absorption efficiency of about 84.7% due to the absorption in the liquid phase (Figure 9). The concentration was maximum in the middle of the tubes and was decreasing in the radial direction of the tubes and along the membrane thickness due to diffusion.

**Figure 10:**
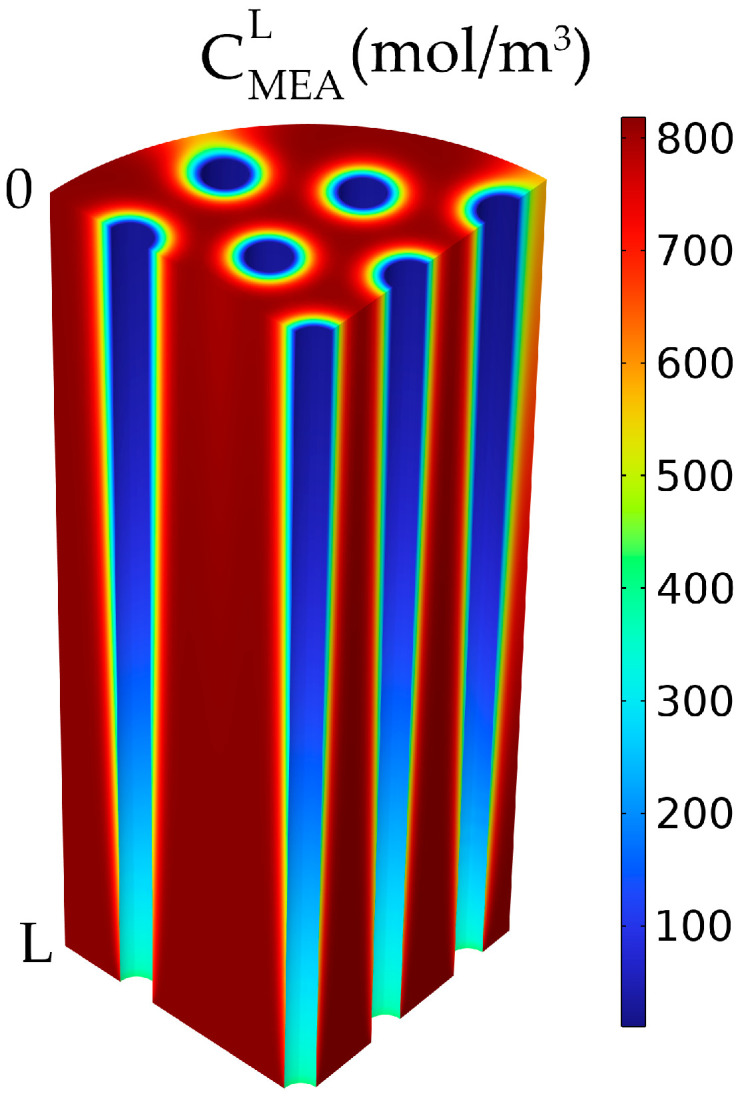
MEA liquid concentration in the shell compartment. $C_{\mathrm{MEA},0}$ = 818 mol/m^3^ (5 wt%), $C_{{\mathrm{CO}}_{2},0}$ = 4.06 mol/m^3^ (10 vol%), Q_g_ = 2 L/min, Q_L_ = 25 L/h.

For the absorbent solution, which flows between the membranes in the shell, the MEA concentration was also decreasing along the membrane length and around the membranes due to the reaction with the absorbed CO_2_ (Figure 10). The MEA concentration decreased along the length of the membranes from 818 mol/m^3^ at the inlet to about 720 mol/m^3^ at the exit of the HFMC. The concentration decreased around the membranes and was lower closer to the membrane walls due to the reaction with the absorbed CO_2_ from the gas phase, while a higher concentration was seen further in the liquid phase, far from the membranes. The absorbed CO_2_ reacted first with the MEA close to the membrane walls, then the CO_2_ diffused inside the liquid phase further into the liquid solution, which resulted in the concentration gradient around the membranes.

### 3.4. Tubes vs. Shell

Two different ways for the flow of the gas mixture and liquid solution were considered inside the HFMC module. 

**Figure 11:**
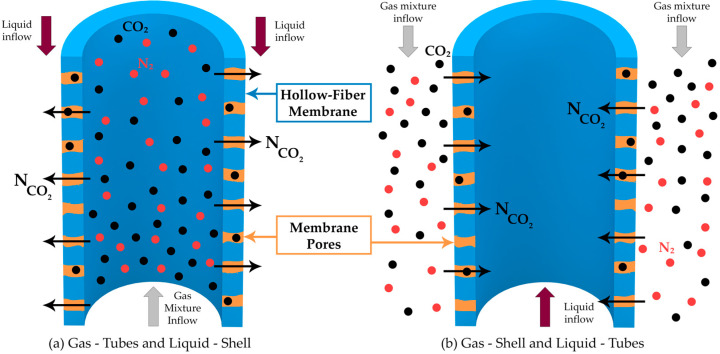
(**a**) Gas mixture inside the membranes (Tubes) and Liquid solution between membranes (Shell), (**b**) Gas mixture between membranes (Shell) and Liquid solution inside the membranes (Tubes).

In the first case, the gas mixture flowed inside the membranes and the liquid MEA solution between them (Figure 11a), while in the second case, the liquid flow was considered inside the membrane and the gas between them (Figure 11b). In both cases, counter-current flow was assumed, with constant flow rates and compositions.

**Figure 12:**
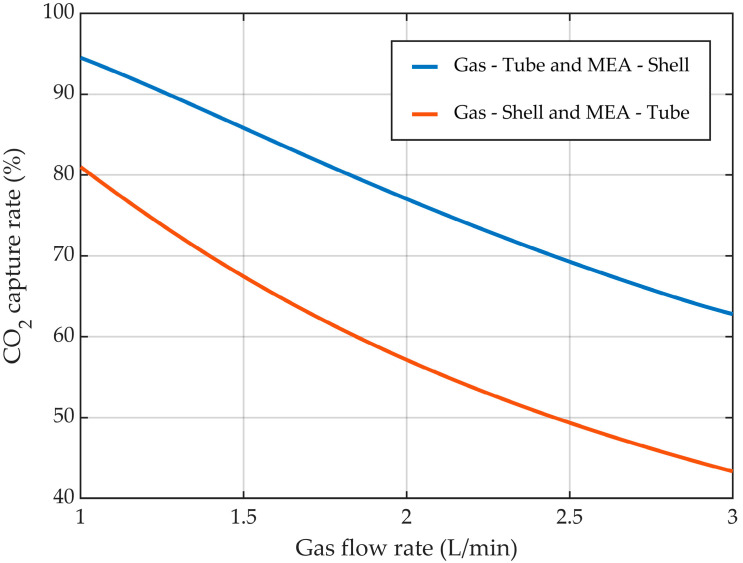
CO_2_ capture rate at different gas flow rates. $C_{\mathrm{MEA},0}$ = 818 mol/m^3^ (5 wt%), $C_{{\mathrm{CO}}_{2},0}$ = 4.06 mol/m^3^ (10 vol%), Q_L_ = 25 L/h.

Regardless of flow configuration (Figure 11), increasing the gas flow rate showed a decrease in the absorption rate of CO_2_ (Figure 12) due to the decrease in the overall residence time for the gas phase. When the gas mixture flowed inside the membranes and the liquid aqueous MEA solution between them (Figure 11a), the absorption efficiency decreased from about 94.5% at a gas flow rate of 1 L/min to 62.8% at a gas flow rate of 3 L/min. In the other case, when the gas mixture passed between the membranes and the liquid absorption solution ran inside them (Figure 11b), the CO_2_ capture rate decreased from 81% at a gas flow rate of 1 L/min to 43.3% at a gas flow rate of 3 L/min. Over the entire range of gas flow variation, the absorption efficiency was considerably higher for the case when the gas mixture flow inside the membranes and the liquid solution between them (Figure 11a). At a gas flow rate of 1 L/min, the difference in absorption efficiency between the two configurations was 13.5%, while at a gas flow rate of 3 L/min, the difference increased to 19.5%.

**Figure 13:**
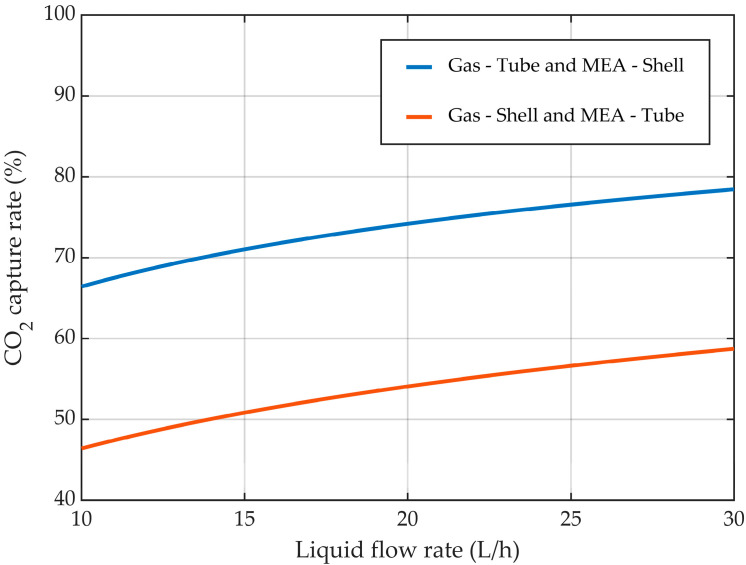
CO_2_ capture rate at different liquid flow rates. $C_{\mathrm{MEA},0}$ = 818 mol/m^3^ (5 wt%), $C_{{\mathrm{CO}}_{2},0}$ = 4.06 mol/m^3^ (10 vol%), Q_g_ = 2 L/min.

The effect of the liquid flow rate considering the two different flow configurations inside the HFMC is represented in Figure 13. The increase in the liquid flow rate resulted in an increase in the CO_2_ capture rate. When the gas mixture flowed inside the membranes and the liquid aqueous MEA solution between them, the absorption efficiency increased from about 66.5% at a liquid flow rate of 10 L/h to nearly 78.5% at a liquid flow rate of 30 L/h. In the other case, when the gas mixture passed between the membranes and the liquid absorption solution inside them, the CO_2_ capture rate increased from 46.5% at a liquid flow rate of 10 L/h to 58.8% at a liquid flow rate of 30 L/h. Along the entire range of investigated liquid flow rates, the absorption efficiency was considerably higher in the case when the gas mixture ran inside the membranes and the liquid solution between them, with a near constant difference between the two cases of around 12.2%.

### 3.5. Membrane Dimensions

An important factor in the absorption process of CO_2_ using HFMC is the dimensions of the membranes, as the mass transfer area between gas and liquid is primarily dictated by the radius of the membrane and the mass transfer resistance through the pores is determined by the thickness of the membrane.

#### 3.5.1. Constant Membrane Thickness

**Figure 14:**
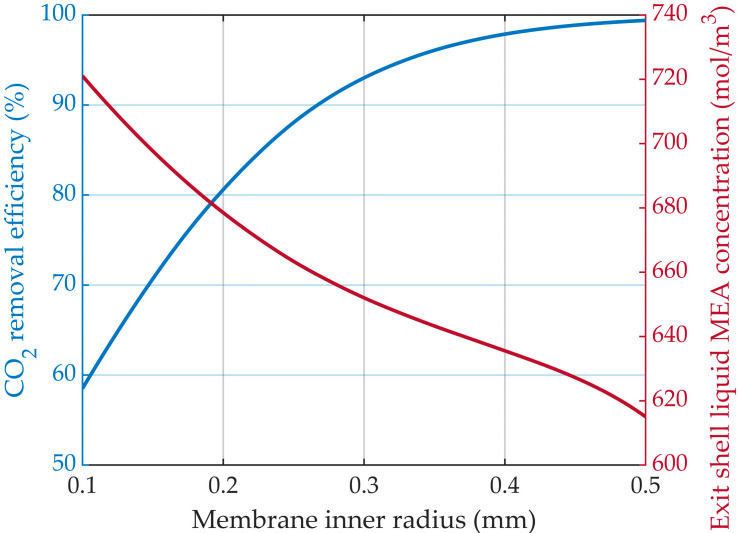
CO_2_ removal efficiency and liquid MEA shell exit concentration profiles at different membrane inner radii and a constant membranes thickness of 0.025 mm. $C_{\mathrm{MEA},0}$ = 818 mol/m^3^ (5 wt%), $C_{{\mathrm{CO}}_{2},0}$ = 4.06 mol/m^3^ (10 vol%), Q_g_ = 2 L/min, Q_L_ = 25 L/h.

The CO_2_ mass transfer resistance through the membrane pores was kept consistent by maintaining a constant membrane thickness. By increasing the membrane radius, the mass transfer area between the gas mixture and liquid solution increased and the gas velocity decreased, resulting in a higher residence time for the gas phase and an increase in the CO_2_ removal efficiency (Figure 14), from around 58.5% for the membrane inner radius of 0.1 mm to nearly 99.5% for a membrane radius of 0.5 mm. 

The liquid MEA concentration decreased from about 720 mol/m^3^ for a membrane inner radius of 0.1 mm to 615 mol/m^3^ for a membrane inner radius of 0.5 mm.

**Figure 15:**
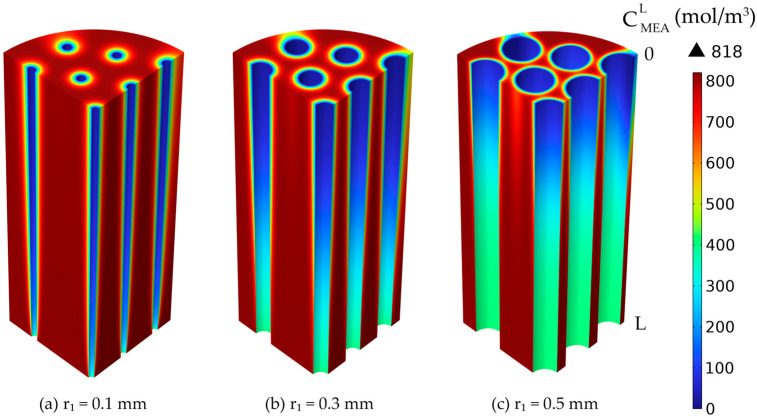
Liquid MEA concentration profiles in the shell compartment, constant membrane thickness 0.025 mm and variable inner radius: (**a**) 0.1 mm, (**b**) 0.3 mm, (**c**) 0.5 mm. $C_{\mathrm{MEA},0}$ = 818 mol/m^3^ (5 wt%), $C_{{\mathrm{CO}}_{2},0}$ = 4.06 mol/m^3^ (10 vol%), Q_g_ = 2 L/min, Q_L_ = 25 L/h.

The MEA concentration decreased along the membrane length and around them due to the reaction with the absorbed CO_2_. The liquid MEA concentration 3D profiles in the liquid phase surrounding the membranes, for different membrane inner radius values but constant thickness, are represented in Figure 15. The increase in the membrane radius led to an increase in the liquid velocity inside the shell compartment of the HFMC.

#### 3.5.2. Variable Membrane Thickness—r_2_ Constant

**Figure 16:**
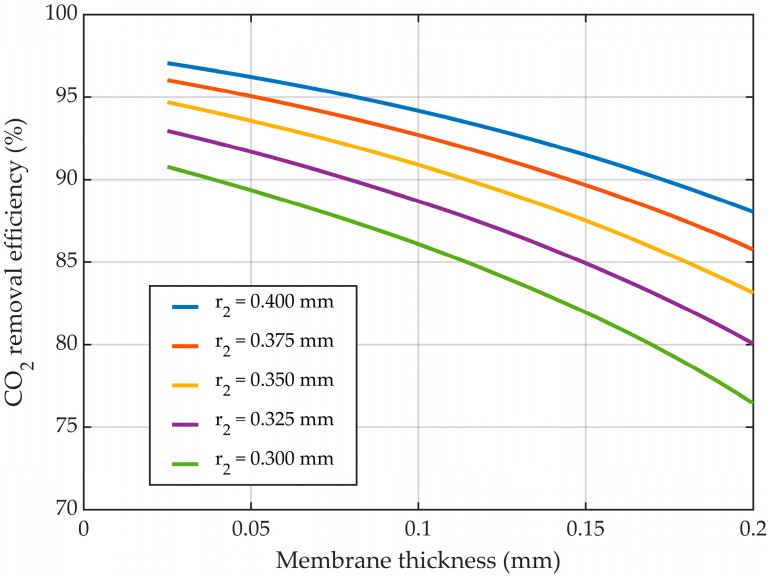
CO_2_ removal efficiency at different membrane thicknesses, outside membrane radius constant, variable inner radius. $C_{\mathrm{MEA},0}$ = 818 mol/m^3^ (5 wt%), $C_{{\mathrm{CO}}_{2},0}$ = 4.06 mol/m^3^ (10 vol%), Q_g_ = 2 L/min, Q_L_ = 25 L/h.

By increasing the membrane thickness, the inner radius decreased, meaning that the velocity for the fluid that ran in the tubes (i.e., the gas mixture) increased, leading to a decrease in the overall residence time of CO_2_ inside the HFMC. In addition, increasing the membrane thickness determined a rise in the mass transfer resistance of CO_2_ through membranes pores. Accounting for both effects, when keeping the membrane outer radius constant, the increase in the membrane thickness led to a decrease in the absorption efficiency of CO_2_ (Figure 16). For the outer membrane radius of 0.3 mm, the CO_2_ removal efficiency was reduced from about 91% at a membrane thickness of 0.025 mm to nearly 76.5% when the membrane thickness was increased to 0.2 mm. By increasing the outer radius of the membranes from 0.3 mm to 0.4 mm, at a constant membrane thickness of 0.1 mm, the absorption efficiency of CO_2_ increased from 86% to about 94.2%, respectively. The change was primarily due to the increase in the mass transfer area between the gas and liquid, but also because of a reduction in gas velocity, thus increasing the residence time of the gas.

**Figure 17:**
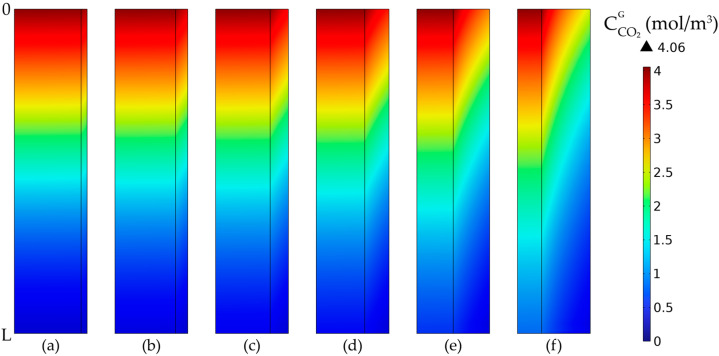
CO_2_ gas concentration profiles in tubes and membranes at a constant outside radius of r_2_ = 0.3 mm and different membrane thicknesses: (**a**) δ = 0.025 mm, (**b**) δ = 0.05 mm, (**c**) δ = 0.075 mm, (**d**) δ = 0.1 mm, (**e**) δ = 0.15 mm, (**f**) δ = 0.2 mm. $C_{\mathrm{MEA},0}$ = 818 mol/m^3^ (5 wt%), $C_{{\mathrm{CO}}_{2},0}$ = 4.06 mol/m^3^ (10 vol%), Q_g_ = 2 L/min, Q_L_ = 25 L/h.

The CO_2_ gas concentration profiles in tubes and membranes at constant outer radius values are presented in Figure 17. The concentration decreased along the length of the membrane due to the absorption in the liquid phase. By increasing the membrane thickness, the mass transfer resistance of CO_2_ through the membranes was increased, thus resulting in an increase in the CO_2_ gradient concentration in the membranes. In addition, by reducing the inner radius of the membranes, the residence time of the gas mixture inside the tubes decreased, resulting in a higher CO_2_ concentration in the exit gas and a lower absorption efficiency.

#### 3.5.3. Variable Membrane Thickness—r_1_ Constant

Maintaining the inner radius of the membrane constant, the mass transfer resistance of CO_2_ through the gas phase was also kept constant. The increase in the membrane thickness led to a higher mass transfer resistance of CO_2_ through the pores of the membrane, as well as an increase in the outer radius of the membranes, which determined an increase in the mass transfer area between the gas mixture and liquid solution. Considering the sum outcome of these two opposite effects on the absorption efficiency of CO_2_, at lower values for the membrane thickness, the effect related to the membrane CO_2_ mass transfer resistance was lower than the effect of the increase in the mass transfer area, resulting in an overall positive effect on the efficiency of absorption. By continuing to increase the membrane thickness, a maximum in CO_2_ removal efficiency was observed at different values of thickness based on the inner radius of the membranes. By further increasing the thickness, the mass transfer resistance of the membranes also increased, leading to a higher overall negative effect on the absorption efficiency.

**Figure 18:**
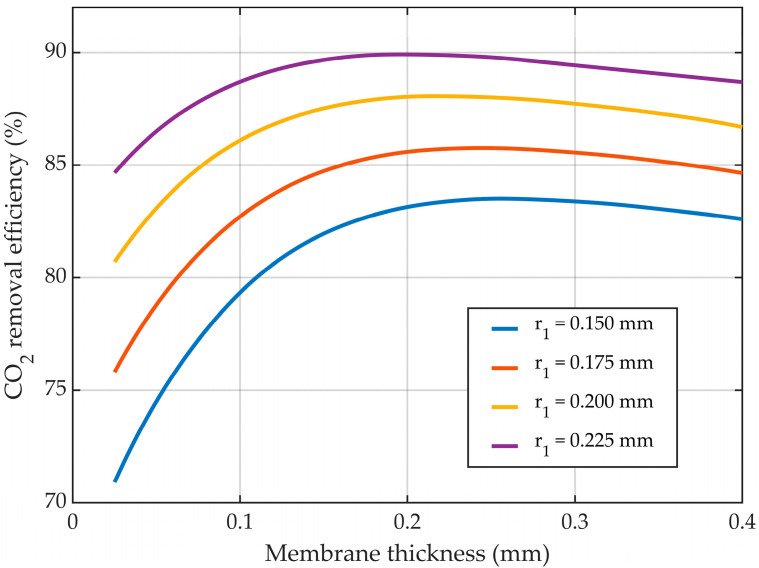
CO_2_ removal efficiency at different membrane thicknesses, for different constant inside radii, and variable outer radii. $C_{\mathrm{MEA},0}$ = 818 mol/m^3^ (5 wt%), $C_{{\mathrm{CO}}_{2},0}$ = 4.06 mol/m^3^ (10 vol%), Q_g_ = 2 L/min, Q_L_ = 25 L/h.

The effect of the membrane thickness for a constant inner radius on the CO_2_ removal efficiency is represented in Figure 18. At a membrane inner radius of 0.2 mm, by increasing the membrane thickness from 0.025 mm to 0.4 mm, the CO_2_ removal efficiency was increased from around 81% to a maximum of about 88% at a thickness of 0.22 mm, after which the removal efficiency decreased to nearly 87%. At different values for the membrane’s inner radius, the maximum values of absorption efficiency were seen at various values for membrane thickness. With increasing inner radius values, the absorption efficiency peaked at lower values for the membrane thickness. For an inner radius of 0.225 mm, the maximum value for the removal efficiency (i.e., nearly 90%) was observed at a thickness of 0.2 mm, while for the lowest inner radius of 0.15 mm, the maximum (i.e., about 83.5%) was noticed at a membrane thickness of around 0.26 mm.

**Figure 19:**
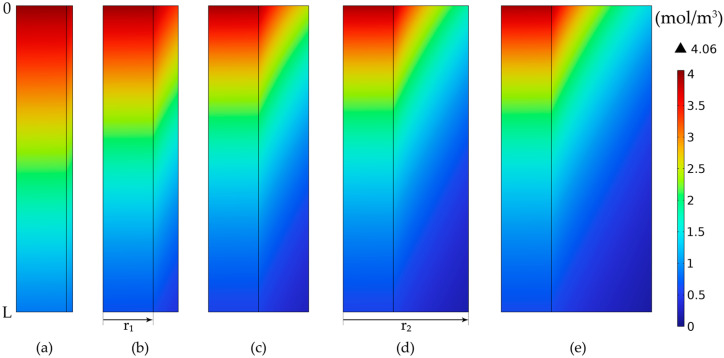
CO_2_ gas concentration profile in tubes and membranes over the membranes’ length and radius, at a constant membrane inside radius r_1_ = 0.2 mm. (**a**) r_2_ = 0.225 mm, (**b**) r_2_ = 0.3 mm, (**c**) r_2_ = 0.4 mm, (**d**) r_2_ = 0.5 mm, (**e**) r_2_ = 0.6 mm. $C_{\mathrm{MEA},0}$ = 818 mol/m^3^ (5 wt%), $C_{{\mathrm{CO}}_{2},0}$ = 4.06 mol/m^3^ (10 vol%), Q_g_ = 2 L/min, Q_L_ = 25 L/h.

The CO_2_ gas concentration profiles in tubes and membranes, for constant inner radius values and different membrane thickness values, are represented in Figure 19. The CO_2_ concentration in the gas phase decreased along the length and radius of the membranes due to the absorption process. By increasing the membrane thickness, the CO_2_ mass transfer resistance was increased together with the concentration gradient inside the membrane.

### 3.6. Membrane Porosity

**Figure 20:**
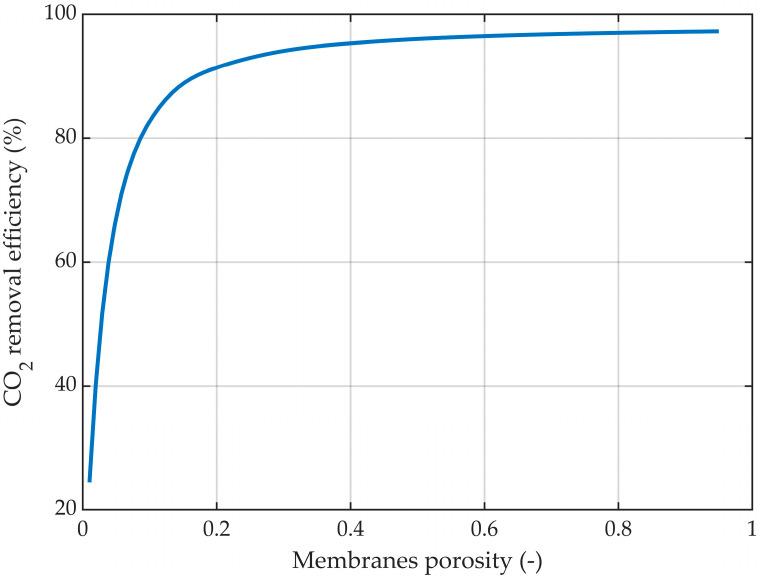
CO_2_ removal efficiency at different membrane porosities. r_1_ = 0.225 mm, δ = 0.2 mm, $C_{\mathrm{MEA},0}$ = 818 mol/m^3^ (5 wt%), $C_{{\mathrm{CO}}_{2},0}$ = 4.06 mol/m^3^ (10 vol%), Q_g_ = 2 L/min, Q_L_ = 25 L/h.

The membrane porosity is an important parameter in the absorption process of CO_2_, with a direct impact on the efficiency of the process. The effect of the membrane porosity on the CO_2_ capture rate is represented in Figure 20.

By increasing the porosity while maintaining constant membrane dimensions, the absorption efficiency increased from about 24.5% at a porosity of 0.01 to around 97.3% at a porosity of 0.95. However, at porosity values lower than 0.2, the increase in the absorption efficiency was considerably higher. The increase from a porosity of 0.01 to 0.2 led to an increase in absorption efficiency of around 67%, while a porosity increase from 0.2 to 1 determined an increase in absorption efficiency of only around 6%.

**Figure 21:**
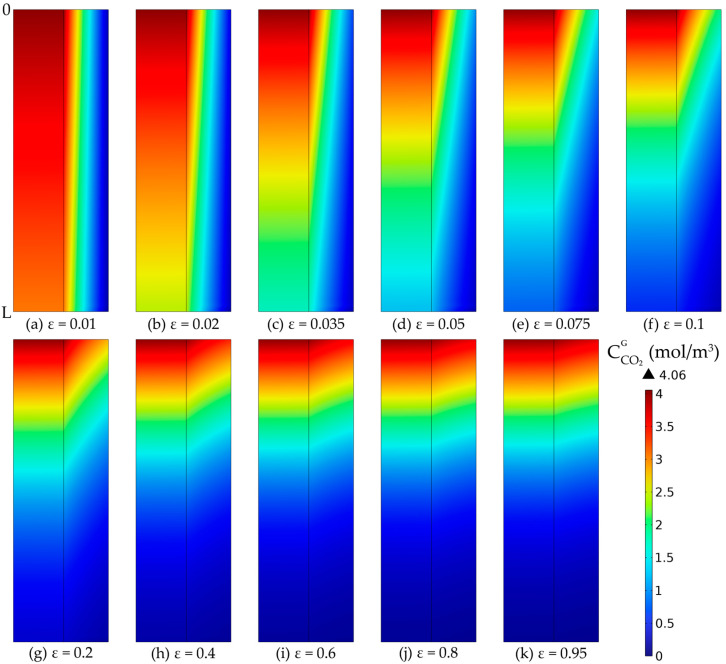
CO_2_ gas concentration profiles in tubes and membranes wall, at a constant membrane radius (r_1_ = 0.225 mm) and thickness (δ = 0.2 mm) and different porosities: (**a**) ε = 0.01, (**b**) ε = 0.02, (**c**) ε = 0.035, (**d**) ε = 0.05, (**e**) ε = 0.075, (**f**) ε = 0.1, (**g**) ε = 0.2, (**h**) ε = 0.4, (**i**) ε = 0.6, (**j**) ε = 0.8, (**k**) ε = 0.95. $C_{\mathrm{MEA},0}$ = 818 mol/m^3^ (5 wt%), $C_{{\mathrm{CO}}_{2},0}$ = 4.06 mol/m^3^ (10 vol%), Q_g_ = 2 L/min, Q_L_ = 25 L/h.

The CO_2_ concentration profiles inside the membrane tube compartments and inside the membranes, at different porosities, are represented in Figure 21, considering the same membrane dimensions in all cases. In all cases, the CO_2_ concentration in the gas phase decreased along the tubes and length and radius of the membranes due to the absorption in the aqueous MEA solution. Because of the diffusion phenomena, a concentration gradient was observed, with the gradient being more pronounced in the membranes compared to the tubes. At a lower porosity, the final concentration of CO_2_ in the purified gases was higher than at a higher porosity, resulting in an improvement in the absorption efficiency with the increase in the membranes’ porosity. At a porosity of 0.01, the CO_2_ concentration in the gas phase at the exit reached nearly 3.07 mol/m^3^ (Figure 21a), but increasing the porosity to 0.1 resulted in a decrease of the exit concentration to around 0.69 mol/m^3^ (Figure 21f), and further increasing the porosity to 0.8 led to a decrease in CO_2_ concentration to around 0.12 mol/m^3^ (Figure 21j).

## 4. Conclusions

A 3D-CFD model of a hollow-fiber membrane contactor for the CO_2_ capture process using an aqueous MEA solution was developed and implemented in COMSOL Multiphysics, considering the entire HFMC geometry. The model described the absorption process of CO_2_ from the gas phase, through the pores of the membrane and into the liquid solution, followed by the chemical reaction with MEA, accounting for both convection and diffusion mechanisms. The model was validated by comparing experimental data published in literature with model predictions on CO_2_ capture rates at different gas and liquid flow rates, as well as different initial concentrations of the flue gases. The simulation results demonstrated excellent correlation with the experimental data, with an R^2^ coefficient value exceeding 0.922. The highest CO_2_ absorption efficiency was achieved at a high liquid flow rate, low gas flow rate, and low CO_2_ concentration in the inflow gas.

Additionally, the optimal mode for fluid flow inside the HFMC was studied, considering identical operating conditions and a counter-current arrangement. The simulation results indicated that superior CO_2_ capture rates were achieved when the gas mixture flowed inside the membranes and the liquid absorption solution ran between them. Furthermore, the effect of the membrane dimensions (i.e., radius and thickness) on the capture process of CO_2_ using HFMC was investigated. Increasing the membrane radius, while maintaining a constant thickness, positively influenced the efficiency of the process due to the higher mass transfer area and longer residence time of the gas phase. On the other hand, higher membrane thickness resulted in higher CO_2_ mass transfer resistance. In order to maximize the absorption efficiency, the findings indicate a low membrane thickness, with optimal dimensions being an inner radius of 0.225 mm and a membrane thickness of 0.2 mm. Additionally, the effect of porosity on the absorption process was investigated, revealing that increasing the porosity led to an improved CO_2_ capture efficiency, but with significant impact only for porosity values below 0.2 and a lesser influence for porosities above that threshold.

## Figures and Tables

**Table 1:** HFMC Polypropylene (PP) module characteristics [31].

Parameters	Value	Unit
Membrane inner radius (r_1_)	0.175	mm
Membrane outer radius (r_2_)	0.2	mm
Number of membranes (n)	510	-
Module inner radius (r_mod_)	1.75	cm
Membrane length (L)	27	cm
Membrane porosity ($\varepsilon_{mem}$)	0.17	-
Membrane tortuosity ($\tau_{mem}$)	19.7	-
Average pores radius (r_p_)	0.05	μm

**Table 2:** Process operating conditions [31].

Parameters	Value	Unit
Gas flow rate (Q_G_)	1–3	L/min
Liquid flow rate (Q_L_)	10–30	L/h
CO_2_ gas concentration ($C_{{\mathrm{CO}}_{2}}$)	10	vol%
MEA liquid concentration (C_MEA_)	5	wt%
Temperature (T)	298	K
Pressure (P)	1	bar

## Data Availability

The original contributions presented in the study are included in the article. Further inquiries can be directed to the corresponding author.

## Nomenclature

*c*	concentration of species (mol/m^3^)
*d*_1_	membrane inside diameter (m)
*d_h_*	hydraulic diameter (m)
*d_lm_*	logarithmic diameter (m)
$D_{j,i},D_{j,mem}$	diffusion coefficient of species j in phase i and membrane (m^2^/s)
*E*	enhancement factor (-)
$H_{CO_{2}}$	Henry’s coefficient of CO_2_ in the liquid MEA solution (-)
$J_{CO_{2}}$	CO_2_ mass transfer flux from gas to liquid (mol/m^2^ s)
$k_{CO_{2},i},k_{CO_{2},mem}$	partial CO_2_ mass transfer coefficient in the i phase and through membrane pores (m/s)
$K_{L}$	global CO_2_ mass transfer coefficient (m/s)
*L*	membranes length (m)
*n*	number of membranes (-)
*P*	pressure (bar)
*Q*	volumetric flow rate (m^3^/s)
*r*_1_	membrane inside radius (m)
*r*_2_	membrane outside radius (m)
*r_mod_*	module inner radius (m)
*r_p_*	average membranes pores radius (m)
$R_{CO_{2}}$	reaction rate (mol/m^3^ s)
*Re*	Reynolds number (-)
*Sc*	Schmidt number (-)
*Sh*	Sherwood number (-)
*T*	temperature (K)
*u*	superficial fluid velocity (m/s)
Superscripts/subscripts	
*i*	gas (g) and liquid (L) phase
*j*	system components
Greek letters	
$\delta_{mem}$	membrane thickness (m)
$\varepsilon_{mem}$	membrane porosity (-)
μ	dynamic viscosity (Pa s)
ρ	fluid density (kg/m^3^)
$\tau_{mem}$	membrane tortuosity (-)
